# Are Stringent Containment and Closure Policies Associated with a Lower COVID-19 Spread Rate? Global Evidence

**DOI:** 10.3390/ijerph19031725

**Published:** 2022-02-02

**Authors:** Zongfeng Xiu, Pengshuo Feng, Jingwei Yin, Yingjun Zhu

**Affiliations:** 1 Business School, Central South University, Changsha 410083, China; xiuzongfeng@csu.edu.cn (Z.X.); fengps@csu.edu.cn (P.F.); 2 School of Accounting, Shanghai Lixin University of Accounting and Finance, Shanghai 201209, China

**Keywords:** government policies, containment and closure, COVID-19, culture, population density

## Abstract

Stringent government policies, in general, and strict containment and closure policies in particular including workplace closing, restrictions on gatherings, close of public transport, stay-at-home order, restrictions on internal movement, and international travel control are associated with a lower spread rate of COVID-19 cases. On the other hand, school closures and public event cancellations have not been found to be associated with lower COVID-19 spread. Restrictions on international travel and the closing of public transport are two policies that stand out and have a consistent and slowing effect on the spread of COVID-19. The slowing effect of the containment and closure policies on the spread of COVID-19 becomes stronger one week after the policies have been implemented, consistent with the SARS-CoV-2 transmission pattern and the incubation period evolution. Furthermore, the slowing effect becomes stronger for culturally tight countries and countries with a higher population density. Our findings have important policy implications, implying that governments need to carefully implement containment and closure policies in their own countries’ social and cultural contexts, with an emphasis on the ideas of the common interest, personal responsibility, and the sense of community.

## 1. Introduction

**Figure 1:**
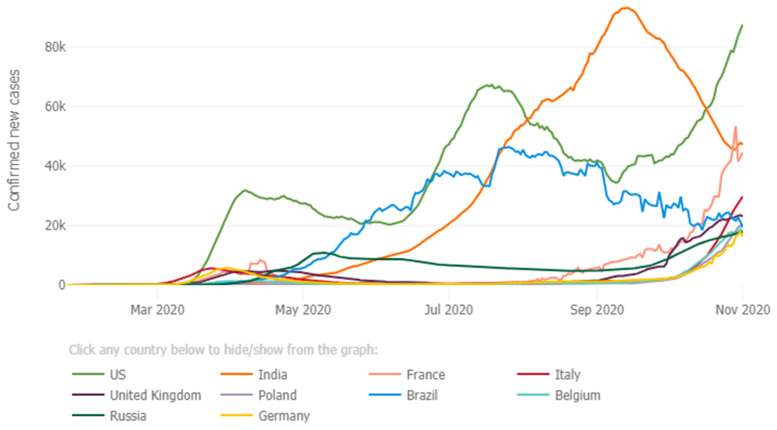
Daily confirmed new COVID-19 cases (5-day moving average) for the current 10 most affected countries (Source: https://coronavirus.jhu.edu/data/new-cases, accessed on 2 November 2020).

SARS-CoV-2, the coronavirus strain responsible for COVID-19, an infectious respiratory disease, has spread rapidly across the world. As of 1 November 2020, COVID-19 has been responsible for more than 47 million cases and a total of 1.2 million deaths worldwide (https://www.worldometers.info/coronavirus/, accessed on 2 November 2020). To combat the quick and widespread nature of COVID-19, governments in many countries around the world have taken a wide range of responses including school and workplace closures, travel restrictions, and stay-at-home orders in an attempt to break the chain of infection [1]. It has been reported that some countries (i.e., South Korea, Japan, and Singapore) are effective in lowering the spread of the disease and reducing cases and deaths [2,3], while some other countries have been badly hit and have witnessed a rampant spread in the virus [4,5]. As shown in Figure 1, given the vast difference in the spread and prevalence of COVID-19 among countries and regions across the world, for example, the first case of COVID-19 in the U.S. was reported on 21 January 2020, but since then, the U.S. has reported 9.2 million cases and more than 230,000 deaths as of 2 November 2020 whereas the first case of COVID-19 in India was reported on 29 January 2020 and the country has reported 8.2 million cases, and more than 122,000 deaths by 2 November 2020; in contrast, the first case was reported on 16 January 2020 in Japan and it has reported 101,813 cases and 1774 deaths by 2 November 2020. In South Korea, the first case was reported on 20 January 2020 and it has reported 26,732 cases and 468 deaths by 2 November 2020 (data obtained from several websites including https://www.worldometers.info/coronavirus/#countries, https://coronavirus.jhu.edu/data/new-cases, and https://en.wikipedia.org/wiki/COVID-19_pandemic_in_South_Korea, accessed on 2 November 2020), a number of natural questions have arisen: what are the important factors associated with the spread of COVID-19? and can stringent government response policies effectively slow down the spread of the virus?

To better understand the transmission pattern of COVID-19 in the very early stages of the pandemic, several scientific studies investigated how transportation convenience [6], demographic characteristics [7,8,9] as well as environmental factors such as temperature, humidity, wind speed, and air pollution levels [7,10,11,12] were associated with the spread and mortality of COVID-19. These studies have reached relatively consistent conclusions. For example, based on the Chinese context, Zhang et al. [6] found that cities that could be easily reached from Wuhan by airports or high-speed trains exhibited a larger number of COVID-19 cases. Li et al. [9] provided global evidence on the argument that people with blood type A had a higher COVID-19 risk compared to other blood types. Regarding environmental factors, Adhikari and Yin [12] found that temperature, humidity, and air pollution were all significantly positively related with new confirmed cases caused by COVID-19, but not significantly related with new deaths resulting from COVID-19 in the U.S. Furthermore, Coskun et al. [7] pointed out that wind speed contributed to the COVID-19 pandemic in Turkey to some extent.

After being aware that COVID-19 is extremely contagious, governments around the world introduced a set of policies to control for the outbreak. As a result, the effects of the above-mentioned factors on the spread of COVID-19 gradually faded as governmental policies played a much more crucial role and outstood their effects [13]. Diao et al. [8] also noted that as the Chinese government implemented a strict lockdown policy at the beginning of the COVID-19 pandemic, there was no association between population density and the COVID-19 spread in China. According to the Oxford COVID-19 Government Response Stringency Index, which are widely used in extant studies [1,14] (refer to the Codebook for the Oxford Covid-19 Government Response Tracker for detailed information, https://github.com/OxCGRT/covid-policy-tracker/blob/master/documentation/codebook.md#codebook-changelog, access on 25 September 2020), governmental policies can be classified into four types: (1) containment and closure policies; (2) economic policies; (3) health system policies; and (4) miscellaneous policies. As for economic and health system polices, a vast body of prior literature has consistently concluded that the amount of economic resource input [9] and medical resource input [9,15,16,17] can both effectively attenuate the COVID-19 pandemic. Specifically, the advocation of hand washing [16] and face mask-wearing [17] were validated to be negatively related with the spread of COVID-19, and hospital bed numbers and COVID-19 test numbers were found to be negatively related with COVID-19 mortality [9,15].

Unlike other types of governmental policies, containment and closure policies have been under considerable debate because it not only erodes civil liberties, but also impairs economic growth [14,18]. Thus, government responses to the COVID-19 pandemic vary dramatically from one country to another, from China’s full-scale lockdown of Wuhan to Sweden’s “never-went-into-lockdown”, and from South Korea’s centralized control and communication [3] to the UK’s “based on the science” approach [19]. Furthermore, empirical evidence for the influence of the stringent containment and closure policies on the spread of COVID-19 is in fact ambiguous. On one hand, some scholars have documented the effectiveness of international travel restrictions [20], the intervention of quarantining [21], and workplace distancing [22] in slowing the spread of COVID-19 within a specific country or region, respectively. On the other hand, Hale et al. [1] noted that the effect of policy responses was highly contingent on local political and social contexts. In this regard, Cepaluni et al. [14] found that stringent containment and closure policies were not effective in mitigating the COVID-19 pandemic in democratic countries. Brodeur et al. [23] found that stay-at-home orders exhibited larger effects in high-trust counties rather than low-trust counties in the U.S. Thus, there is also a call for “smart” stringent measures [18].

In a nutshell, prior literature on stringent containment and closure policies falls short in three aspects, as follows. First, prior literature has examined how different containment and closure policies have affected the spread of COVID-19 [20,21,24], but the resulting evidence has not been assembled and little is known about the total effect of the stringency of containment and closure policies. Second, the majority of studies to conduct empirical analyses have been based on the data of a specific country or region such as China [21], Vietnam [25], Singapore [22], the U.S. [23,26], and so on, and these results cannot be applied to explain variations in the COVID-19 pandemic between countries. Third, there has not been much research focusing on the cross-sectional effects of strict government response policies. As a response, in this study, we seek to fill the void by analyzing the stringency of containment and closure policies on the effect of the spread of COVID-19 in a cross-country setting. In doing so, we aim to contribute to the comprehensive understanding of the effects of the broad, strict government responses and its various aspects, especially in the early stage of the pandemic, when the vaccine for COVID-19 had not been successfully developed yet.

Based on a sample of 6684 observations from 210 countries and regions with reported daily counts of COVID-19 cases from 1 January 2020 to 22 May 2020, we found that strict government response policies were generally associated with a lower spread rate of COVID-19 cases. It is worth noting that we obtained consistent results when using alternative measures of stringency index and after controlling for the potential endogeneity issue. Furthermore, considering the potential lagged effect of the government policies on the new case growth, we used a 1–14 day lagged index in the regression analysis. A negative association between the stringency index and COVID-19 growth rate remained. Notably, there was a downward trend in the magnitude of the coefficients from the first lagged week, with the smallest coefficient for lagged day 7. The coefficient then started to increase on lagged day 8, and eventually fell to the lowest on lagged day 11. The pattern indicates that government policies became the most effective in reducing the COVID-19 spread one week after these policies had been implemented, an important finding consistent with the SARS-CoV-2 transmission pattern’s overall time course for the disease. That is, once an individual is exposed and becomes infected, the incubation period before the onset of symptoms is about five days, with day 7 being the worst. After day 11, most patients who survive are on their way to recovery [27].

Government policies cannot be implemented in a vacuum. Cultural factors have been found to influence people’s behavior, political participation, economic activities, and social norms and values [28]. Government policies are more likely to be acceptable to the public and achieve desirable outcomes if policymakers take culture and values into consideration [29], as collective actions from individuals eventually determine the effectiveness of government policies. Culture is defined by Hofstede [30] as “the collective programming of the mind that distinguishes the members of one category of people from others”, and it has several important dimensions. We further our study by considering one dimension of culture across different countries: cultural tightness–looseness and examine its effect on the association between government policy stringency and the spread of COVID-19. A country’s culture is oriented toward tightness if it has many strong norms and low tolerance of deviant behaviors. In contrast, a country’s culture is considered loose if it has weak social norms and high tolerance for deviant behavior [31,32]. The result shows that the slowing effect of the government’s strict containment and closure policies on the spread of COVID-19 becomes stronger for culturally tight countries than for culturally loose countries. This finding implies that in order to suppress the spread of the virus, governments need to carefully implement policies in their own social and cultural context.

Several studies have shown that population density aggravates the spread of COVID-19 [33,34,35] as more face-to-face interactions among people tend to take place in dense areas, which makes the rapid spread of cases easier in populated areas. We proceeded to investigate the effect of a country’s population density on the negative effect of containment and closure policies on the spread of COVID-19. After classifying the countries into high- vs. low-populated subsamples, we reported that the negative effect of the government’s strict policies on the spread of COVID-19 became stronger for highly populated countries than less populated countries.

Overall, our findings have two critically important policy implications. First, considering the rampant spread of COVID-19 cases as well as the most recent COVID-19 surge in the U.S. and several European countries, we recommend that governments need to carefully consider their containment and closure policies, as some policies are more closely associated with a lower spread of COVID-19 cases while other policies are not. In particular, six out of eight indicators of containment and closure policies were found to be associated with a lower spread rate of COVID-19 cases including workplace closure, restrictions on gatherings, closure of public transport, stay-at-home requirements, restrictions on internal movement and international travel controls, while two policies—school closures and cancellation of public events—were not associated with a lower spread of COVID-19. After including all eight indicators into one single regression and examining their effects simultaneously on the spread of COVID-19, two policies dominated and had a negative association with the spread of COVID-19: closure of public transport and restrictions on international travel. Second, we also recommend that governments carefully implement policies within their own countries’ social and cultural contexts, as the negative association between government containment and closure policies and the spread of COVID-19 becomes stronger for countries with a tight culture. This finding implies that to suppress the spread of the virus, the governments might want to implement public health policies that emphasize the ideas of the common interest, personal responsibility, and the sense of a community, as people all live together in a small global village.

## 2. Data, Samples, and Variables

We collected the daily COVID-19 case data for the major countries around the world from the Our World in Data website (https://ourworldindata.org/), where the goal of the website is to make the knowledge on large problems accessible and understandable. COVID-19 data used in the study was downloaded from https://github.com/owid/covid-19-data/tree/master/public/data on 22 May 2020. After excluding the data that had no new cases reported on initial days and that had missing control variables in the regression analysis (control variables as specified below), we obtained a final sample of 6684 observations for 210 countries with reported daily counts of COVID-19 cases from 1 January 2020 to 22 May 2020. As we began this research on 22 May 2020, we chose the sample period from 1 January 2020 to 22 May 2020. Moreover, this study primarily aimed to contribute to the comprehensive understanding of the effects of the broad strict government responses on the spread of COVID-19, especially in the early stage of the pandemic, when the vaccine for COVID-19 had not yet been successfully developed. Thus, the sample period from 1 January 2020 to 22 May 2020 is appropriate to some extent. In fact, countries or regions around the world started to vaccinate people for COVID-19 beginning the end of 2020. To further ensure our conclusions, we re-conducted analyses based on an expanded sample covering 1 January 2020 to 31 December 2020. Our untabulated results revealed that the main conclusions were still valid after the sample period being expanded to 31 December 2020. Overall, the findings are useful for governments to take appropriate measures in the event of the next pandemic. We downloaded the data for the expanded sample on 15 January 2022 and the tables are available from the authors upon request. Two key variables in the data are (1) COVID-19 daily new case growth rate or *CASES_GROW*; and (2) government policy stringency index or *STRINGENCY_INDEX*, a measure indicating how strict a country’s government containment and closure policies are in response to the spread of COVID-19 cases. To be specific, the new case growth rate for a country *i* on a day *t* is calculated as follows: *CASES_GROW_i,t_ = (NEW_CASES_i,t_ − NEW_CASES_i,t−_*_1_*)/NEW_CASES_i,t−_*_1_. Government policy stringency index or *STRINGENCY_INDEX* represents a composite measure and is rescaled to a value from 0 to 100 (100 = the strictest response). It is necessary to state that we did not calculate the government responses stringency index (i.e., *STRINGENCY_INDEX*) by ourselves, and that the data on *STRINGENCY_INDEX* was directly obtained from https://www.bsg.ox.ac.uk/research/research-projects/covid-19-government-response-tracker on 22 May 2020 (i.e., the Oxford COVID-19 Government Response Tracker, Blavatnik School of Government). We also derived three alternative measures for government stringency policy: *STRINGENCY_TERCILE_i,t_, STRINGENCY_MEDIAN_ADJUSTED_i,t_,* and *STRINGENCY_STANDARDIZED_i,t_*. To be specific, we created a category variable *STRINGENCY_TERCILE_i,t_* by splitting the sample into three groups based on the original stringency index of a government *i* and assigned a measure of 1, 2, and 3, if its stringency index is in the bottom, medium, and top terciles of all sample countries on a certain day *t*, corresponding to three different strictness levels of a government’s policy response to the COVID-19 spread: below average, average, and above average. *STRINGENCY_MEDIAN_ADJUSTED_i,t_* is a country’s stringency index adjusted by all the country’s median levels of the index on a day *t*. *STRINGENCY_STANDARDIZED_i,t_* is a normalized index by considering both the mean and standard deviation of the original stringency index on day *t* (note here the day t refers to the *t*th day since a country reports its first case of COVID-19, not the calendar day). Finally, considering the potential lagged effect of the government policies on the new case growth, we also used a 1–14 day lagged index in the analysis.

Other control variables in the regression analysis include the following: *LN_POPULATION*, *POPULATION_DENSITY*, *LN_MEDIAN_AGE*, *AGED_70_OLDER*, *LN_GDP_PER_CAPITA*, *LN_CVD_DEATH_RATE*, *DIABETES_PREVALENCE*, *MALE_SMOKERS*, *FEMALE_SMOKERS*, and *HOSPITAL_BEDS_PER_100K*. The definitions of these control variables and data sources are provided in Appendix A.

Table 1 reports the descriptive statistics of the main variables used in the study. All the continuous variables were winsorized at 1% and 99%. It is worth noting that our conclusions were still valid after all the continuous variables being winsorized at 1.5% and 98.5% (tables are available from the authors upon request). The average (median) spread rate of COVID-19 *(CASES_GROW)* was 22.41% (0%), with a standard deviation of 125.37%. The maximum spread rate on a particular day was as high as 788.24%. The average number of new cases (*NEW_CASES*) was 437.3359. Government policy stringency index (*STRINGENCY_INDEX*) had a mean (median) value of 71.4082 (79.4900), with a standard deviation of 23.8832. The minimum and maximum values were zero and 100, suggesting that the governments in the sample implemented dramatically different policies as a response to the COVID-19 global pandemic. The average for *STRINGENCY_TERCILE, STRINGENCY_MEDIAN_ADJUSTED*, and *STRINGENCY_STANDARDIZED* was 2.1142, −4.6471, and 0.0273, respectively. The average of *LN_MEDIAN_AGE* was 3.5258. The average (median) number of people over a land area in square kilometers or population density (*POPULATION_DENSITY*) in the sample was 286.1975 (97.9990), ranging from 3.0780 to 7915.7310 with a standard deviation of 983.7277. On average, approximately 7.3985% of the population in the sampling countries were 70 years and older, as measured by *AGED_70_OLDER*. There were about 3.4920 hospital beds per 100,000 people in our sample, with a minimum and maximum of 0.3000 and 13.05 beds per 100,000 people. On average, there were 198.86 deaths from cardiovascular disease in 2017 every year per 100,000 people, and about 7.7999% of the population aged 20 to 79 in 2017 suffered from diabetes. Finally, as measured by *MALE_SMOKERS* and *FEMALE_SMOKERS*, the percent of smokers in the male and female population accounted for about 31.8790% and 11.8606%, respectively.

## 3. Empirical Results

### 3.1. Government Stringent Responses and COVID-19 Spread

We constructed the following regression model to investigate the association between government policy stringency and the spread of COVID-19 cases.

*CASES_GROW_i,t_* = *α* + *β*_1_
*STRINGENCY_INDEX_i,t_* + *β*_2_
*LN_POPULATION_i,t_*

*β*_3_*POPULATION_DENSITY_i,t_* + *β*_4_
*LN_MEDIAN_AGE_i,t_*

*β*_5_*AGED_70_OLDER_i,t_* + *β*_6_
*LN_GDP_PER_CAPITA_i,t_*

*β*_7_*LN_CVD_DEATH_RATE_i,t_* + *β*_8_
*DIABETES_PREVALENCE_i,t_*

*β*_9_*MALE_SMOKERS_i,t_* + *β*_10_
*FEMALE_SMOKERS_i,t_*

*β*_11_*HOSPITAL_BEDS_PER_100K_i,t_* + *WEEK FIXED EFFECTS*

+ *COUNTRY FIXED EFFECTS* (1)

In addition to the original government response stringency index or *STRINGENCY_INDEX*, we used *STRINGENCY_TERCILE_i,t_*, *STRINGENCY_MEDIAN_ADJUSTED_i,t_*, and *STRINGENCY_STANDARDIZED_i,t_* as alternative independent variables in the regression analysis.

We first estimated the correlation coefficients among the main variables used in the regression analysis. Our untabulated results showed that *CASES_GROW* had a negative and significant correlation with all four government response stringency indexes (we did not tabulate the correlation matrix to preserve the space. The table is available from the authors upon request). In particular, the correlations between *CASES_GROW* and *STRINGENCY_INDEX, STRINGENCY_TERCILE, STRINGENCY_MEDIAN_ADJUSTED,* and *STRINGENCY_STANDARDIZED* were −0.0411, −0.0422, −0.0460, and −0.0358, respectively, all significant at a 1% significance level. There was a positive correlation between *CASES_GROW* and *DIABETES_PREVALENCE* (*ρ =* 0.0208, *p <* 0.1). We did not find a severe issue of multicollinearity among the independent variables.

We conducted an ordinary least square (OLS) regression after controlling for country-level and weekly fixed effects, whereas the standard errors were clustered at a country level. As reported in Table 2, all four measures of government stringency indexes had a negative and significant coefficient across four regression specifications, indicating that strict government response policies are generally associated with a slow spread rate of COVID-19 cases. In particular, the coefficient of *STRINGENCY_INDEX* in column (1) was negative and significant (*β* = −0.0043, *t* = −3.22) after controlling for several factors, implying that for one standard deviation increase in the stringency index (σ = 23.8832, as reported in Table 1), the COVID-19 spread rate decreases approximately by 10.27%. Given the average daily new cases was about 437.3359 in our sample (reported in Table 1), this is equivalent to a reduction of 44.91 new cases on a daily basis. Consistently, the coefficient of *STRINGENCY_TERCILE* is also negative and coefficient (*β* = −0.0752, *t* = −3.50). The result indicates that for every one level increase of strictness of government response to COVID-19 cases (i.e., from a “below average” to “average” or from an “average” to “above average”), the COVID-19 spread rate decreased approximately by 7.52%, equivalent to a reduction of 32.89 new cases daily. Another two alternative measures, *STRINGENCY_MEDIAN_ADJUSTED* and *STRINGENCY_STANDARDIZED* both had a negative and significant coefficient, as shown in columns (3) and (4), providing consistent evidence to support a negative association between government stringent policies and the COVID-19 spread rate. We also investigated the influence of government stringent policies on the COVID-19 death rate. DEATH_RATE is defined as the ratio of total COVID-19 deaths to total COVID-19 cases on a certain date. Our untabulated results showed that the coefficients on *STRINGENCY_INDEX*, *STRINGENCY_TERCILE*, *STRINGENCY_MEDIAN_ADJUSTED* and *STRINGENCY_STANDARDIZED* were all insignificant, indicating that although government stringent policies play an important role in slowing down the spread of COVID-19, they cannot effectively mitigate the COVID-19 mortality (the table is available from the authors upon request).

Regarding the control variables in the regression results in Table 2, we note some important findings pertinent to the factors that are associated with the spread of COVID-19. Generally speaking, the COVID-19 cases spread quickly in countries with a higher population density, more people older than 70, more people that had developed diabetes, and more hospital beds. To be specific, *POPULATION_DENSITY* had a positive and significant coefficient with a 1% significance level across the four regression specifications. Take column (3), for instance, its coefficient was 0.0095, implying that every 10 people increase in a nation’s population density is associated with an increase of 9.5% of COVID-19 cases. *AGED_70_OLDER* had a positive and significant coefficient, ranging from 0.5380 in column (4) to 0.5725 in column (2). A 1% increase in the percentage of the population older than 70 was related to an increase of 0.5380–0.5725% new cases of COVID-19 every day. The percentage of the population that had developed with diabetes was another important factor influencing the COVID-19 spread, as indicated by a positive and significant coefficient of *DIABETES_PREVALENCE* across the four regression models. A 1% increase in the percentage of the population aged 20 to 79 who had diabetes was related to an increase of 0.5882–0.6701% of new cases of COVID-19 on a daily basis. Somewhat surprisingly, *HOSPITAL_BEDS_PER_100K* had a positive effect on the spread of COVID-19. The reason could be due to the increased hospital capacity to admit more COVID-19 cases. Wealthy countries, measured by *LN_GDP_PER_CAPITA*, generally had a lower spread rate of COVID-19 cases, as shown by the negative and significant coefficients of *LN_GDP_PER_CAPITA*. A country’s populations’ median age or *LN_MEDIAN_AGE*, the death rate from cardiovascular disease or *LN_CVD_DEATH_RATE*, and the percentage of women and men who smoked or *FEMALE_SMOKERS* and *MALE_SMOKERS* were all negatively and significantly related to the spread rate of COVID-19 cases. Dowd et al. [36] showed that older people were associated with a higher fatality rate of COVID-19. We here report that countries with a population of younger median age are associated with a lower spread rate of COVID-19. Overall, Table 2 shows that stringent government response policies are associated with a lower spread rate of COVID-19. In addition, COVID-19 cases spread more quickly in countries with an older population, heavier population density, more people with diabetes, and a lower national income.

### 3.2. Containment and Closure Policies and the COVID-19 Spread

Our results thus far are based on a board government policy stringency index, which includes eight indicators: (1) school closing; (2) workplace closing; (3) cancellation of public events; (4) restrictions on gatherings; (5) public transportation closing; (6) stay-at-home order; (7) restrictions on internal movement; and (8) international travel controls. In this section, we regressed the growth rate of COVID-19 cases against the eight indicators to examine which containment and closure policy, in particular, was attributed to a lower spread rate of COVID-19. We included the same set of control variables in the regression as in Table 2.

Table 3 reports the results. As described in detail in Appendix B, each category was coded on an ordinal scale that represents the level of strictness of a certain policy. Among the eight specific containment and closure policies, we found that school closures and cancellation of public events had no significant effects on reducing the COVID-19 spread; while the other six policies including workplace closing, restrictions on gatherings, the closing of public transport, stay-at-home requirements, restrictions on internal movement, and international travel controls were significantly and negatively associated with the lower COVID-19 spread, as indicated by their negative and significant coefficients in columns (2), and (4)–(8), respectively. In addition to their statistical significance, the economic significance of these specific policies is sizable, as interpreted in detail below.

1. Regarding the government policies on workplace closure, *WORKPLACE_CLOSING* had a coefficient of −0.0785 (*t* = −3.45), as shown in column (2). The result indicates that for every one level escalation of strictness in response to COVID-19 cases, that is, (i) from “no measures” to “recommend work from home”, or (ii) from “recommend work from home” to “require work from home for some sectors or categories of workers”, or (iii) from “require work from home for some sectors or categories of workers” to “require work from home for all-but-essential workplaces”, the COVID-19 spread rate decreased approximately by 7.85% daily. Given that the average daily new cases was about 437.3359 in our sample (reported in Table 1), this is equivalent to a reduction of 34.33 new cases on a daily basis. The effect would be even greater if the policy is escalated to the highest level of “require work from home for all-but-essential workplaces” from the lowest level of “no measures”. That is, 102.99 new cases would have been reduced daily in our sampling countries.
2. Regarding the government policies on restrictions on gatherings, the result in column (4) implies that for every one level increase of strictness in response to COVID-19 cases, the COVID-19 spread rate decreased approximately by 5% daily, equivalent to a reduction of 21.87 new cases daily. The effect would be even greater if the policy is escalated to the highest level of “restrictions on gatherings of 10 people or less” from the lowest level of “no restrictions”. That is, 87.47 new cases would have been reduced daily in our samples.
3. As reported in column (5), regarding the government policies on closing public transportation, for every one level increase of strictness in response to COVID-19 cases, the COVID-19 spread rate decreased approximately by 15.21% daily, equivalent to a reduction of 66.52 new cases daily. The effect would be even greater if the policy is escalated to the highest level of “require closing (or prohibit most citizens from using it)” from the lowest level of “no restrictions”. That is, 133.04 new cases would have been reduced daily in the sampling countries.
4. Column (6) shows the result of government policies on the stay-at-home requirement. For every one level increase of strictness in response to COVID-19 cases, the COVID-19 spread rate decreased approximately by 11.02% daily, equivalent to a reduction of 48.19 new cases daily. The effect would be even bigger if the policy is escalated to the highest level of “require not leaving the house with minimal exceptions (e.g. allowed to leave once a week, or only one person can leave at a time, etc.)” from the lowest level of “no restrictions”. That is, 144.58 new cases would have been reduced daily in our sample.
5. Regarding the government policies on domestic travel, the result in column (7) indicates that for every one level increase of strictness in response to COVID-19 cases, the COVID-19 spread rate decreased by approximately 9.75% daily, equivalent to a reduction of 42.64 new cases daily. The effect would be even greater if the policy is escalated to the highest level of “internal movement restrictions in place” from the lowest level of “no restrictions”. That is, 85.28 new cases would have been reduced daily in the sample.
6. As shown in column (8), government policies on international travel were also associated with COVID-19 spread. For every one level increase of strictness in response to COVID-19 cases, the COVID-19 spread rate decreased by approximately 8.13% daily, equivalent to a reduction of 35.56 new cases daily. The effect would be even greater if the policy is escalated to the highest level of “ban on all regions or total border closure” from the lowest level of “no restrictions”. That is, 142.22 new cases would have been reduced daily in our sampling countries.

**Table 3:** Regression analysis of the effect of containment and closure policies on the COVID-19 spread.

Variables	(1)	(2)	(3)	(4)	(5)	(6)	(7)	(8)	(9)
*SCHOOL_CLOSING*	−0.0261								0.0196
	(−0.86)								(0.60)
*WORKPLACE_CLOSING*		−0.0785 ***							−0.0228
		(−3.45)							(−0.78)
*CANCEL_PUBLIC_EVENTS*			−0.0323						0.0831
			(−0.65)						(1.39)
*RESTRICTIONS_ON_GATHERINGS*				−0.0500 ***					−0.0220
				(−2.64)					(−1.01)
*CLOSE_PUBLIC_TRANSPORT*					−0.1521 ***				−0.0976 **
					(−3.99)				(−2.51)
*STAY_AT_HOME_REQUIREMENTS*						−0.1102 ***			−0.0509
						(−4.05)			(−1.55)
*RESTRICTIONS_ON_INTERNAL_MOVEMENT*							−0.0975 ***		−0.0093
							(−2.87)		(−0.23)
*INTERNATIONAL_TRAVEL_CONTROLS*								−0.0813 ***	−0.0644 **
								(−2.95)	(−2.17)
*LN_POPULATION*	−0.0108 *	0.0302 **	−0.0101	0.0220	0.0212 *	0.0098	−0.0115 *	−0.0197 ***	0.0413 *
	(−1.96)	(2.21)	(−1.61)	(1.54)	(1.80)	(1.32)	(−1.96)	(−3.13)	(1.68)
*POPULATION_DENSITY*	0.0108 ***	0.0094 ***	0.0106 ***	0.0102 ***	0.0098 ***	0.0098 ***	0.0104 ***	0.0100 ***	0.0089 ***
	(79.97)	(21.86)	(34.59)	(38.75)	(30.93)	(32.92)	(54.31)	(33.27)	(16.65)
*AGED_70_OLDER*	0.5620 ***	0.5804 ***	0.5515 ***	0.6081 ***	0.6218 ***	0.5951 ***	0.5605 ***	0.5286 ***	0.6348 ***
	(59.45)	(67.20)	(37.61)	(33.06)	(39.50)	(59.52)	(81.78)	(41.75)	(17.44)
*LN_MEDIAN_AGE*	−7.2584 ***	−7.0737 ***	−7.0568 ***	−7.9806 ***	−7.2911 ***	−7.3770 ***	−7.0144 ***	−6.5031 ***	−7.4625 ***
	(−83.15)	(−72.85)	(−22.53)	(−29.80)	(−97.57)	(−97.32)	(−57.75)	(−24.04)	(−11.78)
*LN_GDP_PER_CAPITA*	−1.2989 ***	−1.0633 ***	−1.2899 ***	−1.0221 ***	−1.0922 ***	−1.0983 ***	−1.3117 ***	−1.4881 ***	−1.0178 ***
	(−41.15)	(−15.59)	(−54.12)	(−9.97)	(−20.55)	(−22.54)	(−72.21)	(−21.07)	(−5.22)
*LN_CVD_DEATH_RATE*	−0.5560 ***	−0.2637 ***	−0.5378 ***	−0.2365**	−0.1501	−0.2394 ***	−0.5176 ***	−0.7659 ***	−0.1070
	(−26.79)	(−3.33)	(−51.34)	(−2.09)	(−1.56)	(−3.24)	(−42.08)	(−9.86)	(−0.49)
*DIABETES_PREVALENCE*	0.6505 ***	0.5839 ***	0.6395 ***	0.6096 ***	0.6067 ***	0.6040 ***	0.6389 ***	0.6655 ***	0.5929 ***
	(70.76)	(30.10)	(61.79)	(39.08)	(51.42)	(49.02)	(106.82)	(80.30)	(18.34)
*MALE_SMOKERS*	−0.0776 ***	−0.0701 ***	−0.0767 ***	−0.0684 ***	−0.0728 ***	−0.0715 ***	−0.0770 ***	−0.0802 ***	−0.0683 ***
	(−61.34)	(−31.35)	(−100.59)	(−19.66)	(−49.86)	(−44.06)	(−107.25)	(−67.05)	(−12.90)
*FEMALE_SMOKERS*	−0.0516 ***	−0.0727 ***	−0.0498 ***	−0.0680 ***	−0.0819 ***	−0.0664 ***	−0.0536 ***	−0.0532 ***	−0.0949 ***
	(−15.84)	(−10.39)	(−19.04)	(−9.49)	(−10.15)	(−15.04)	(−19.96)	(−22.04)	(−7.77)
*HOSPITAL_BEDS_PER_100K*	0.2336 ***	0.1789 ***	0.2258 ***	0.2070 ***	0.1795 ***	0.1687 ***	0.2222 ***	0.2541 ***	0.1706 ***
	(50.52)	(11.45)	(32.55)	(20.69)	(12.55)	(10.58)	(47.04)	(32.27)	(6.03)
*CONS*	33.2319 ***	28.6568 ***	32.4776 ***	30.7495 ***	28.9014 ***	29.9807 ***	32.4805 ***	34.0489 ***	28.3462 ***
	(54.11)	(21.05)	(45.59)	(31.03)	(24.47)	(34.18)	(75.34)	(68.80)	(12.94)
*WEEK FIXED EFFECTS*	YES	YES	YES	YES	YES	YES	YES	YES	YES
*COUNTRY FIXED EFFECTS*	YES	YES	YES	YES	YES	YES	YES	YES	YES
N	6411	6395	6390	6391	6369	6392	6368	6389	6316
R^2^	0.0302	0.0310	0.0291	0.0303	0.0321	0.0316	0.0309	0.0315	0.0358

This table reports the regression results of the association between a specific government policy: the containment and closure measures and the COVID-19 spread rate. The containment and closure measures include eight indicators: (1) school closing, (2) workplace closing, (3) cancellation of public events, (4) restrictions on gatherings, (5) public transportation closing, (6) stay-at-home order, (7) restrictions on internal movement, and (8) international travel controls. The policy measures data were obtained from the Oxford COVID-19 Government Response Tracker, Blavatnik School of Government. The dependent variable is the new case growth rate for a country *i* on a day *t*, or *CASES_GROW*. The Appendix A provides the definitions of the main variables as well as data resources. All reported *t*-values in parentheses are based on standard errors adjusted for country-level clustering. The symbols of ***, ** and * represent the 1%, 5%, and 10% levels of significance, respectively, for a two-tailed test.

We included all eight policy measures into a single regression in column (9) and examined their effects simultaneously on the spread of COVID-19. We found that two policies eventually dominated: the closure of public transportation and restrictions on international travel. These results demonstrate a critically important role in closing public transport and restricting international travel in slowing down the spread of COVID-19.

### 3.3. A Lagged Effect of Government Policies on the COVID-19 Spread

There may be a time lag between the government implementing policies as a response to the COVID-19 spread. That is, when the government takes actions by adopting strict prevention and control policies, it may take several days before the spread of COVID-19 cases starts to decrease. This is particularly likely considering that SARS-CoV-2, the virus that causes COVID-19, has been found to have a 14-day incubation period—the time between an individual being exposed to the virus and when they start to develop symptoms. Thus, we lagged *STRINGENCY_INDEX* by 1–14 days, respectively, to test the robustness of our previous findings (using the lagged *STRINGENCY_INDEX* in the regression can also mitigate the potential endogeneity issue, as discussed in Section 3.6).

**Figure 2:**
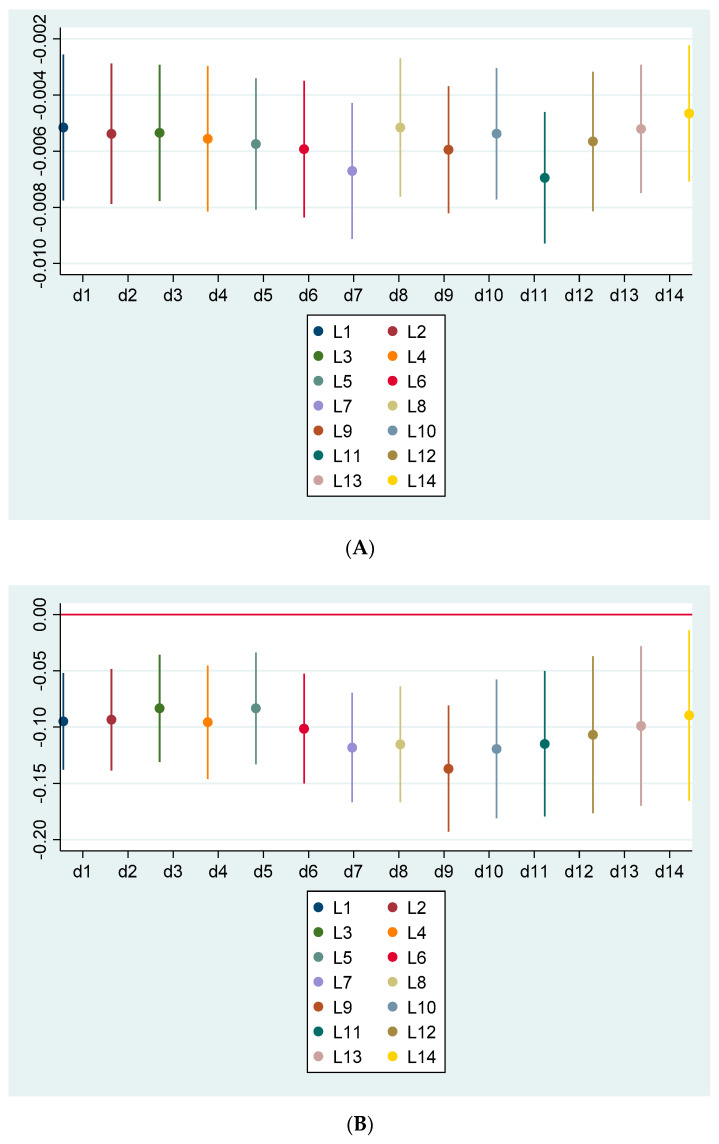

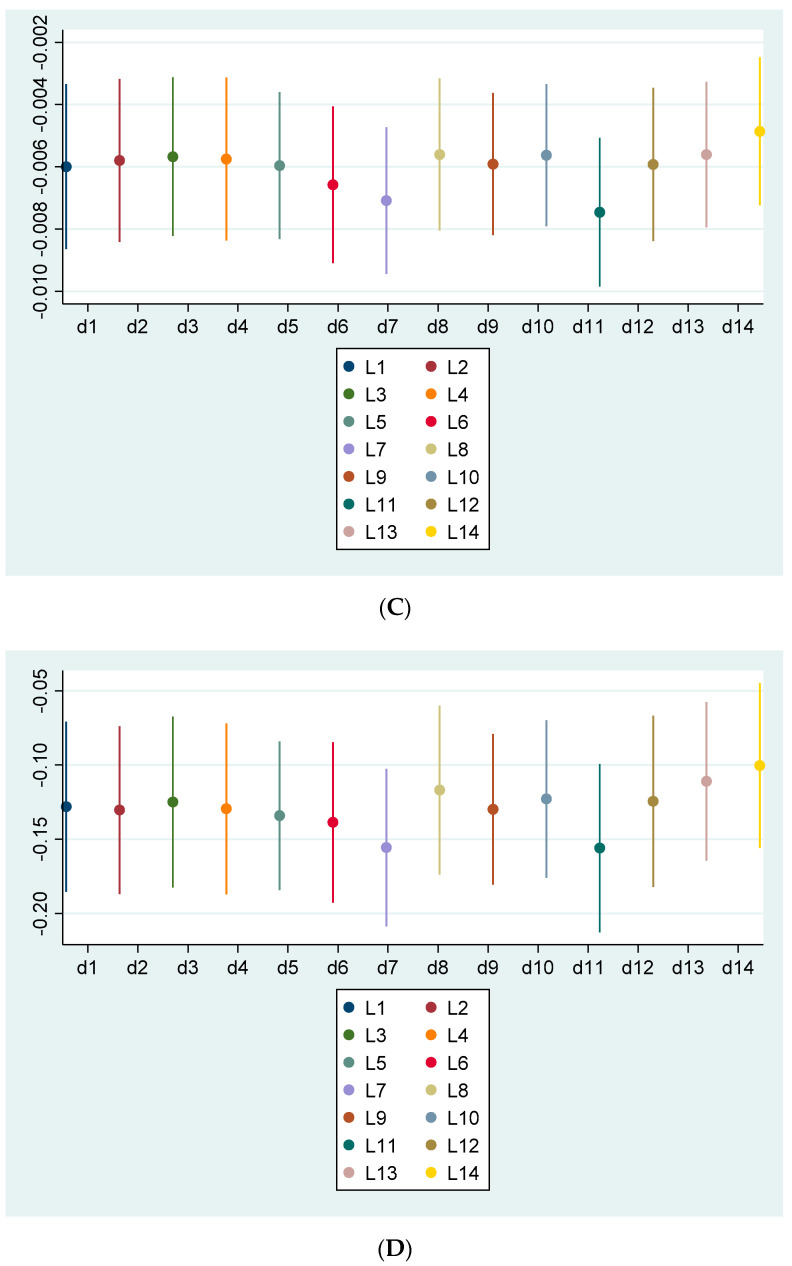
Lagged effect of government responses on the COVID-19 spread. Panel (**A**). Plot of coefficients of the original government stringency index (*STRINGENCY_INDEX*); Panel (**B**). Plot of coefficients of the government stringency index classified into the bottom, medium, and top terciles (*STRINGENCY_TERCILE*); Panel (**C**). Plot of coefficients of the government stringency index adjusted by the median level (*STRINGENCY_MEDIAN_ADJUSTED*); Panel (**D**). Plot of coefficients of normalized government stringency index (*STRINGENCY_STANDARDIZED*).

We continued to find that all the coefficients of the lagged stringency index, from *LAG_1DAY_STRINGENCY_INDEX* to *LAG_14DAY_STRINGENCY_INDEX* were negative and significant, further confirming the important negative association between the strictness of a government response and the COVID-19 spread rate. That is, the more stringent the government response policies, the lower the COVID-19 spread rate. The results on the control variables were generally consistent with those reported in Table 2. After plotting the coefficients of these 14-lagged indexes in Figure 2, panel A, where *d1*, *d2…d14* indicates the coefficient of 1-, 2-, … 14-day lagged government stringency index, respectively, we first observed a downward trend of the magnitude of the coefficients from the first lagged week, with the smallest coefficient of the lagged day 7 (*β* = −0.0067). The coefficients then started to increase on the lagged day 8 and gradually dropped to the lowest level on lagged day 11 (*β* = −0.0069). The pattern in Figure 1 shows that government response policies start to exert the largest effect on the COVID-19 spread one week after these policies have been implemented. This finding is consistent with the SARS-CoV-2 transmission pattern for the disease. Once a person is exposed to the virus and is infected, the incubation period before the onset of symptoms is about five days, with the worst symptoms appearing on day 7. After day 11, most patients are on their way to recovery [27].

We next lagged the other three alternative measures, *STRINGENCY_TERCILE, STRINGENCY_MEDIAN_ADJUSTED,* and *STRINGENCY_STANDARDIZED* by 1–14 days and conducted the regression analysis again. We plotted the coefficients of the lagged indexes in Figure 2. All coefficients of government stringency indexes were negative and significant, with an identical pattern of Figure 1 remaining. Overall, we report robust and consistent results that strict government response policies are associated with a lower spread rate of COVID-19 cases.

### 3.4. Cultural Tightness vs. Looseness

Cultures guide the individual’s decisions, actions, and social behaviors at an unconscious level [37]. They also play an important role in shaping government policies. Government policies are more likely to be acceptable to the public and achieve desirable outcomes if governments take cultures and values into consideration [29]. In this section, we analyzed the effect of national cultures on the association between government policy stringency and the spread of COVID-19 from one aspect of the culture: cultural tightness–looseness. According to Gelfand et al. [31] and Harrington and Gelfand [32], cultural tightness–looseness assesses how much a culture adheres to social norms and tolerates deviance. A tight culture is restrictive, has many norms, and takes strict disciplinary actions for the violation of norms, while a loose culture has few and relaxed social norms and a high tolerance for deviant behaviors. Countries with tight cultures tend to strictly enforces rules and discourage individualistic thinking or behavior, while countries with loose cultures give people more freedom in how they behave and what they believe in. As a tight culture allows people to coordinate more effectively to survive threats and natural disasters [31,32] and is associated with increased government control and constraints in daily life [38], we expect that the effect of government stringent policies on the COVID-19 spread will be stronger in countries and regions that are more oriented toward cultural tightness.

We obtained the cultural tightness–looseness scores for 33 countries and regions from Gelfand et al. [31] and merged them with the COVID-19 data. We used the 30% and 60% quantile, corresponding to the score of 5.4 and 7, respectively, as a cutoff to classify a country or region as culturally tight- vs. loose-oriented. In particular, countries and regions with a score equal to or less than 5.4 were classified as a LOOSE group, while countries and regions with a score equal to or above 7 as a TIGHT group. The LOOSE group includes Australia, Brazil, Estonia, Greece, Hungary, Israel, the Netherlands, Spain, Ukraine, New Zealand, the United States and the TIGHT group includes China, Germany, India, Japan, Malaysia, Mexico, Norway, Pakistan, Portugal, Singapore, Turkey, and South Korea. We also employed t-tests to examine the differences in the mean values of the major variables between the TIGHT and LOOSE groups. Our untabulated results showed that there was no significant difference in the mean value of CASES_GROW between the two groups, indicating that cultural tightness does not affect the spread of COVID-19 directly and it would be appropriate for us to further investigate whether the effects of strict government response policies on the spread of COVID-19 is contingent on regional cultural features (the table is available from the authors upon request). We then conducted the original regressions using these two subsamples and report the results in Table 4. As columns (1) and (2) show, the estimated coefficient of *STRINGENCY_INDEX* in the LOOSE subsample was −0.0038 (*t* = −1.48), and −0.0105 in the TIGHT subsample (*t* = −2.95). We obtained similar and consistent results when using other alternative measures of government stringent policy indexes (for instance, when using the median-adjusted government stringency index (*STRINGENCY_MEDIAN_ADJUSTED*), the estimated coefficient of *STRINGENCY_MEDIAN_ADJUSTED* in the LOOSE subsample was −0.0046 (*t* = −1.68), and −0.0107 in the TIGHT subsample (*t* = −3.03)). The magnitude of differences in the coefficients was sizable—the coefficient of government stringency indexes in the TIGHT group was approximately 2.76 times that in the LOOSE group (−0.0105 vs. −0.0038). The result shows that the slowing effect of stringent government policies on COVID-19 spread is much stronger in countries or regions with tight cultures than in countries or regions with loose cultures. Martin and Yoon [3] reported that South Korea was about twice as effective as the U.S. and UK at preventing infected individuals from spreading the disease to others, according to a recent report from a United Nations-affiliated research network. However, we acknowledge that our result here does not necessarily imply that when a country or region is more toward a tight culture, its governmental strict policies in response to the COVID-19 become 2.76 times more effective in slowing down the spread of COVID-19 than in other countries or regions more toward a loose culture. The purpose of the paper was not to assess the effectiveness of certain government policies. Instead, we aimed to investigate the association between government stringent policies and the COVID-19 spread rate.

### 3.5. Population Density

Previous findings in Table 2 showed a positive relation between *POPULATION_DENSITY* and the COVID-19 growth rate as more face-to-face interactions among people tend to take place in dense areas, making it likely in populated areas to suffer a rapid spread of cases [33]. In the U.S., lower population density was found to be associated with a decrease in the instantaneous reproduction number of SARS-CoV-2, the coronavirus strain responsible for COVID-19 [34]. Similar finding of a strong correlation between the population density and the number of COVID-19 infected cases has been reported in countries such as Algeria [35] and Turkey [7]. In this section, we investigate whether the effect of a country’s government stringent policies on the spread of COVID-19 varies with the population density.

We used the 20% and 80% quantiles, corresponding to the number of people per square kilometers, 25.04 and 266.886, respectively, as a cutoff to classify countries and regions in the sample as most- vs. least-populated. In particular, countries with a population density equal to or less than 25.04 were classified as a LEAST group while countries with a population density equal to or above 266.886 as a MOST group. (The LOW group includes Algeria, Argentina, Australia, Botswana, Brazil, Canada, Chile, Finland, Iceland, Kazakhstan, Mali, Mongolia, New Zealand, Niger, Norway, Oman, Paraguay, Russia, Saudi Arabia, Suriname, Sweden, Uruguay, and Zambia. The HIGH group includes Bahrain, Bangladesh, Barbados, Belgium, El Salvador, India, Israel, Japan, Lebanon, Mauritius, the Netherlands, Philippines, Singapore, South Korea, Sri Lanka, the United Kingdom, and Vietnam). We then conducted the original regression using these two subsamples and report the results in Table 5. As columns (1) and (2) show, the estimated coefficient of *STRINGENCY_INDEX* in the LEAST subsample was −0.0016 (*t* = −0.49), and −0.0060 in the MOST subsample (*t* = −3.09). We obtained similar and consistent results when using other alternative measures of government stringent policy indexes (for instance, when using the median-adjusted government stringency index (*STRINGENCY_MEDIAN_ADJUSTED*), the estimated coefficient of *STRINGENCY_MEDIAN_ADJUSTED* in the LEAST subsample was −0.0034 (*t* = −1.01), and −0.0060 in the MOST subsample (*t* = −2.92)). The magnitude of differences in the coefficient was sizable—the coefficient of government stringency indexes in the MOST group was approximately 3.75 times that in the LEAST group (−0.0060 vs. −0.0016). The result shows that when a country or region is highly populated, the effect of its government’s strict policies on the COVID-19 spread becomes stronger than a less populated country or region.

### 3.6. Endogeneity Issue

A potential issue related to the previous results is endogeneity. In the early stages of the COVID-19 outbreak, the growth rate of positive cases in a country tends to be high. At this time, the governments are likely to adopt more stringent prevention and control measures to flatten the curve. If this is possible, it is natural to find a negative association between strict policies and the COVID-19 growth rate. As we have shown in Section 3.3, using the lagged *STRINGENCY_INDEX* (from day 1 to day 14) in the regressions can help mitigate the potential endogeneity issue. In this section, we adopt an instrumental variable (IV) method to alleviate the endogeneity concern. Another source of endogeneity is omitted unobservable variables that affect both dependent and independent variables, so we used the country level and weekly fixed effect to mitigate this concern. In particular, we used the Freedom in the World Index (*FREEDOM_INDEX*) as an IV (the Freedom in the World Index ranks political rights and civil liberties for most of the countries in the world. The index construction process can be accessed at: https://freedomhouse.org/reports/freedom-in-the-world/freedom-in-the-world-research-methodology, accessed on 22 May 2020) and conducted a two-stage least square regression. We expected a significant and negative association between a country’s *FREEDOM_INDEX* and the stringency of government response policies, as democratic countries are positioned to develop better policies to emerging issues and public health crises than other authoritarian counterparts due to their enhanced information transparency, capacities, and trust from the general public [39,40]. Meanwhile, as an infectious respiratory disease, COVID-19 has spread quickly across almost all countries and regions worldwide, regardless of democratic or authoritarian regimes. As column (1) of Table 6 shows, the coefficient of the *FREEDOM_INDEX* was significant and negative at the 1% level (*β* = −16.5872, *t* = −75.13) and the Kleibergen–Paaprk Wald *F*-statistic was 143.838, higher than the 10% critical value of 16.38, indicating that the instrument is a valid measure in the first stage of the regression. Consistent with our expectations, this result shows that democratic governments tend to implement less strict policies in response to the COVID-19 pandemic. When using the predicted stringency index (*STRINGENCY_INDEX_PREDICTED*) as the independent variable in column (2), we found that it had a negative and significant coefficient (*β* = −0.0353, *t* = −111.30), implying that a negative association between the stringency of government policies and COVID-19 spread is robust after controlling for the potential endogeneity issue.

## 4. Conclusions

Overall, we conducted the first comprehensive study on the effect of government containment and closure policies on the COVID-19 spread. We found that the stringent policies were generally associated with the lower spread rate, and such a negative association became stronger one week after these policies had been implemented (or on day 7), for culturally tight countries and for countries with a higher population density. To shed more light on the effects of governmental policies on the spread of COVID-19, we regressed the growth rate of COVID-19 cases against the eight specific policies to examine which aspects of the broad stringency index mattered the most for the lower spread of COVID-19. Our regression analysis showed that among several government policies, workplace closure, restrictions on gatherings, closure of public transport, stay at home requirements, restrictions on internal movement and international travel controls were associated with a lower spread rate of COVID-19 cases while school closure and canceling public events were not. After including all policies into one single regression and examining their effects simultaneously on the COVID-19 spread, we found that two policies dominated and had a negative association with the spread of COVID-19: closure of public transport and restrictions on international travel. These findings have important policy implications and indicate that to slow down the spread of COVID-19, governments need to carefully consider their containment and closure policies.

This study also had its limitations. First, our sample period only covered the early stage of the COVID-19 pandemic (i.e., the period without an effective vaccine for COVID-19). However, although a great number of countries (regions) around the world have started to vaccinate people against COVID-19 since the end of 2020, the constantly mutating COVID-19 virus brings a series of new challenges to governments and makes the situation more and more complicated. Due to the limitation of our sample period, we have no idea about whether stringent government policies are still effective in slowing the spread rate of COVID-19 in such different situations. Thus, we highly recommend that future research can better answer this question. Second, it is suggested that stringency may have some negative effects on a society along with its positive effect on the control of the COVID-19 pandemic [14,18]. However, this study primarily focused on the bright side of stringent government containment and closure policies, and provided little evidence on the dark side of it. In this regard, it is necessary for future research to develop a comprehensive index to capture the overall influence of stringency on a society. Finally, this study found that stringent government containment and closure policies exhibited stronger effects for countries (regions) with a higher level of cultural tightness and population density. It would be appropriate for future research to investigate whether its effects are also contingent on other national features, and how different aspects of government policies (i.e., containment and closure policies, economic policies, health system policies, and miscellaneous policies) interactively affect the spread of COVID-19.

## Figures and Tables

**Table 1:** Descriptive statistics.

Variables	N	Mean	SD	Min	P25	P50	P75	Max
*CASES_GROW*	6684	0.2241	1.2537	−1.0000	−0.3750	0.0000	0.3813	7.8824
*NEW_CASES*	6684	437.3359	1001.4436	0.0000	8.0000	50.0000	295.0000	4805.0000
*STRINGENCY_INDEX*	6684	71.4082	23.8832	0.0000	61.2400	79.4900	88.7500	100.0000
*STRINGENCY_TERCILE*	6684	2.1142	0.7937	1.0000	1.0000	2.0000	3.0000	3.0000
*STRINGENCY_MEDIAN_ADJUSTED*	6684	−4.6471	20.9930	−83.6000	−12.4400	0.3325	8.3300	67.6000
*STRINGENCY_STANDARDIZED*	6675	0.0273	0.9297	−4.3526	−0.3909	0.2835	0.6828	2.4794
*LN_POPULATION*	6684	16.6855	1.6737	11.4962	15.5129	16.6859	17.8121	21.0454
*POPULATION_DENSITY*	6684	286.1975	983.7277	3.0780	46.7540	97.9990	214.2430	7915.7310
*AGED_70_OLDER*	6684	7.3985	4.5071	0.6170	3.2620	6.9380	11.5800	16.2400
*LN_MEDIAN_AGE*	6684	3.5258	0.2578	2.7973	3.3776	3.5723	3.7377	3.8691
*LN_GDP_PER_CAPITA*	6684	9.8069	1.0346	6.6238	9.2669	10.0331	10.5904	11.4540
*LN_CVD_DEATH_RATE*	6684	5.2926	0.5085	4.4515	4.8542	5.2939	5.6289	6.3920
*DIABETES_PREVALENCE*	6684	7.7999	3.6503	1.9100	5.5000	7.1100	9.5900	22.0200
*MALE_SMOKERS*	6684	31.8790	12.6190	8.5000	21.4000	30.9000	40.8000	76.1000
*FEMALE_SMOKERS*	6684	11.8606	10.4430	0.2000	1.9000	7.8000	20.0000	35.3000
*HOSPITAL_BEDS_PER_100K*	6684	3.4920	2.6515	0.3000	1.6000	2.7700	4.5100	13.0500

This table reports descriptive statistics for the major variables used in the study. Daily COVID-19 case data for 210 countries worldwide from 1 January 2020 to 22 May 2020 were obtained from the Our World in Data website. Government policy stringency index data were from the Oxford COVID-19 Government Response Tracker, Blavatnik School of Government. Appendix A provides the definitions of main variables as well as data resources.

**Table 2:** Regression analysis of the effect of government policy stringency index on the COVID-19 spread.

Variables	(1)	(2)	(3)	(4)
*STRINGENCY_INDEX*	−0.0043 ***			
	(−3.22)			
*STRINGENCY_TERCILE*		−0.0752 ***		
		(−3.50)		
*STRINGENCY_MEDIAN_ADJUSTED*			−0.0052 ***	
			(−3.72)	
*STRINGENCY_STANDARDIZED*				−0.1166 ***
				(−3.93)
*LN_POPULATION*	−0.0192 ***	−0.0160 ***	−0.0216 ***	−0.0288 ***
	(−4.06)	(−3.26)	(−4.54)	(−4.73)
*POPULATION_DENSITY*	0.0097 ***	0.0114 ***	0.0095 ***	0.0097 ***
	(27.22)	(50.29)	(26.07)	(31.64)
*AGED_70_OLDER*	0.5510 ***	0.5725 ***	0.5497 ***	0.5380 ***
	(75.38)	(81.11)	(75.81)	(58.09)
*LN_MEDIAN_AGE*	−6.8121 ***	−7.5662 ***	−6.7360 ***	−6.6900 ***
	(−41.74)	(−66.63)	(−41.10)	(−39.80)
*LN_GDP_PER_CAPITA*	−1.1774 ***	−1.3147 ***	−1.1569 ***	−1.1710 ***
	(−27.26)	(−77.69)	(−26.02)	(−29.38)
*LN_CVD_DEATH_RATE*	−0.3711 ***	−0.4990 ***	−0.3360 ***	−0.3615 ***
	(−6.64)	(−30.72)	(−5.74)	(−7.26)
*DIABETES_PREVALENCE*	0.5974 ***	0.6701 ***	0.5882 ***	0.5912 ***
	(34.13)	(82.90)	(32.89)	(36.02)
*MALE_SMOKERS*	−0.0727 ***	−0.0795 ***	−0.0719 ***	−0.0722 ***
	(−41.98)	(−87.33)	(−40.32)	(−43.31)
*FEMALE_SMOKERS*	−0.0604 ***	−0.0458 ***	−0.0622 ***	−0.0572 ***
	(−16.28)	(−17.22)	(−16.42)	(−20.87)
*HOSPITAL_BEDS_PER_100K*	0.2110 ***	0.2481 ***	0.2069 ***	0.2129 ***
	(26.77)	(40.74)	(25.03)	(31.33)
*CONS*	30.3346 ***	33.9560 ***	29.6910 ***	29.8882 ***
	(29.74)	(78.37)	(27.39)	(29.92)
*WEEK FIXED EFFECTS*	YES	YES	YES	YES
*COUNTRY FIXED EFFECTS*	YES	YES	YES	YES
N	6684	6684	6684	6675
R^2^	0.0309	0.0299	0.0318	0.0318

This table reports the OLS regression results of the association between the government policy stringency index and the COVID-19 spread rate. The dependent variable is the new case growth rate for a country *i* on a day *t*, or *CASES_GROW*, calculated as follows: *CASES_GROW_i,t_ = (NEW_CASES_i,t_-NEW_CASES_i,t−_*_1_*)/ NEW_CASES_i,t−_*_1_. The Appendix A provides the definitions of the main variables as well as the data resources. All reported *t*-values in parentheses are based on standard errors adjusted for country-level clustering. The symbols of *** represent the 1% level of significance, for a two-tailed test.

**Table 4:** Regression analysis of the effect of cultural tightness–looseness on the correlation between government responses and the COVID-19 spread.

Variables	(1) LOOSE	(2) TIGHT
*STRINGENCY_INDEX*	−0.0038	−0.0105 **
	(−1.48)	(−2.95)
*LN_POPULATION*	0.0772	0.0635 ***
	(1.18)	(7.27)
*POPULATION_DENSITY*	0.0005 **	−0.0000 ***
	(2.81)	(−3.31)
*AGED_70_OLDER*	0.2777 *	−0.0092
	(2.13)	(−1.23)
*LN_MEDIAN_AGE*	−6.0024 *	1.1522 ***
	(−2.19)	(4.61)
*LN_GDP_PER_CAPITA*	−0.2915 **	−0.1471 **
	(−2.73)	(−2.29)
*LN_CVD_DEATH_RATE*	0.4367 *	−0.0993
	(2.02)	(−0.92)
*DIABETES_PREVALENCE*	−0.0095	−0.0230 ***
	(−0.60)	(−5.01)
*MALE_SMOKERS*	−0.0219	0.0099 **
	(−1.18)	(3.01)
*FEMALE_SMOKERS*	0.0223	−0.0011
	(1.46)	(−0.29)
*HOSPITAL_BEDS_PER_100K*	−0.0265 ***	−0.0772 ***
	(−4.36)	(−4.52)
*CONS*	19.0952 **	−2.8299 *
	(2.39)	(−2.03)
*WEEK FIXED EFFECTS*	YES	YES
*COUNTRY FIXED EFFECTS*	YES	YES
N	798	1009
R^2^	0.0398	0.0512

This table reports the regression results of the association between a country’s cultural tightness vs. looseness and the COVID-19 spread rate. The cultural tightness–looseness scores for 33 countries were obtained from Gelfand et al. [31] and then merged with the COVID-19 data from 1 January 2020 to 22 May 2020. We used the 30% and 60% quantiles, corresponding to the score of 5.4 and 7, respectively, as a cutoff to classify a country as culturally tight- vs. loose-oriented. In particular, countries with a score equal to or less than 5.4 were classified as a LOOSE group while countries with a score equal to or above 7 as a TIGHT group. We then conducted the regression analysis for these two subsamples, respectively. The dependent variable is new case growth rate for a country *i* on a day *t*, or *CASES_GROW*. The Appendix A provides the definitions of main variables as well as data resources. All reported *t*-values in parentheses were based on standard errors adjusted for country-level clustering. The symbols of ***, **, and * represent the 1%, 5%, and 10% levels of significance, respectively, for a two-tailed test.

**Table 5:** Regression analysis of the effect of population density on the correlation between government responses and the COVID-19 spread.

Variables	(1) Least	(2) Most
*STRINGENCY_INDEX*	−0.0016	−0.0060 ***
	(−0.49)	(−3.09)
*LN_POPULATION*	−0.0174	−0.2599 ***
	(−0.59)	(−4.12)
*POPULATION_DENSITY*	0.1173 ***	−0.0000 ***
	(10.80)	(−3.80)
*AGED_70_OLDER*	−0.3395 ***	0.0537 ***
	(−5.37)	(3.44)
*LN_MEDIAN_AGE*	5.0990 ***	−1.1509
	(4.48)	(−1.33)
*LN_GDP_PER_CAPITA*	−0.8176***	0.1703
	(−4.17)	(0.94)
*LN_CVD_DEATH_RATE*	−1.2313 ***	0.6281
	(−3.24)	(1.00)
*DIABETES_PREVALENCE*	−0.0125 ***	−0.0251
	(−3.63)	(−1.11)
*MALE_SMOKERS*	0.0082 ***	−0.0048
	(3.62)	(−1.42)
*FEMALE_SMOKERS*	0.0412 ***	−0.0242 ***
	(3.40)	(−5.61)
*HOSPITAL_BEDS_PER_100K*	0.1875 **	0.0289 *
	(2.28)	(1.91)
*CONS*	−3.0251 ***	3.9827
	(−2.87)	(0.56)
*WEEK FIXED EFFECTS*	YES	YES
*COUNTRY FIXED EFFECTS*	YES	YES
N	1286	1155
R^2^	0.0348	0.0492

This table reports the regression results of the association between a country’s population density and the COVID-19 spread rate. We used the 20% and 80% quantile, corresponding to the number of people per square kilometers, 25.04 and 266.886, respectively as a cutoff to classify countries in the sample as the most- vs. least-populated. In particular, countries with a population density equal to or less than 25.04 were classified as the LEAST group while countries with a population density equal to or above 266.886 as the MOST group. We then conducted the regression analysis for these two subsamples, respectively. The dependent variable was the new case growth rate for a country *i* on a day *t*, or *CASES_GROW*. The Appendix A provides the definitions of main variables as well as data resources. All reported *t*-values in parentheses were based on standard errors adjusted for country-level clustering. The symbols of ***, **, and * represent the 1%, 5%, and 10% levels of significance, respectively, for a two-tailed test.

**Table 6:** Endogeneity check using the country Freedom in the World Index as an instrumental variable.

Variables	(1) STRINGENCY_INDEX	(2) CASES_GROW
*FREEDOM_INDEX*	−16.5872 ***	
	(−75.1304)	
*STRINGENCY_INDEX_PREDICTED*		−0.0353 ***
		(−111.2981)
*LN_POPULATION*	−39.9735 ***	−0.1015 ***
	(−114.0754)	(−18.9480)
*POPULATION_DENSITY*	−0.6830 ***	0.0025 ***
	(−68.3462)	(46.4547)
*AGED_70_OLDER*	194.4369 ***	0.4856 ***
	(82.5026)	(78.9334)
*LN_MEDIAN_AGE*	−1905.1409 ***	−3.6214 ***
	(−79.4920)	(−64.8338)
*LN_GDP_PER_CAPITA*	165.9606 ***	−0.4306 ***
	(59.5568)	(−37.5322)
*LN_CVD_DEATH_RATE*	−303.1061 ***	0.8499 ***
	(−65.5361)	(51.9923)
*DIABETES_PREVALENCE*	29.9748 ***	0.2453 ***
	(99.0861)	(114.7384)
*MALE_SMOKERS*	2.9409 ***	−0.0424 ***
	(43.8874)	(−95.6032)
*FEMALE_SMOKERS*	−17.6555 ***	−0.1311 ***
	(−91.9176)	(−61.5783)
*HOSPITAL_BEDS_PER_100K*	16.1073 ***	0.0740 ***
	(77.8955)	(27.6377)
*CONS*	6894.5887 ***	11.6673 ***
	(84.8811)	(56.7092)
*WEEK FIXED EFFECTS*	YES	YES
*COUNTRY FIXED EFFECTS*	YES	YES
N	6684	6684
R^2^	0.6727	0.0287

This table reports the 2-stage regression results of the association between a government policy stringency index and the COVID-19 spread rate using the Freedom in the World Index or *FREEDOM_INDEX* as an IV. The dependent variable is *STRINGENCY_INDEX* in column (1) while the dependent variable is the COVID-19 spread rate (*CASES_GROW*) in column (2). The predicted value of the government stringency index, or *STRINGENCY_INDEX_PREDICTED* is used in column (2). The Appendix A provides the definitions of the main variables as well as data resources. All reported *t*-values in parentheses were based on standard errors adjusted for country-level clustering. The symbols of ***, **, and * represent the 1%, 5%, and 10% levels of significance, respectively, for a two-tailed test.

## Data Availability

Publicly available datasets were analyzed in this study. This data can be found here: https://www.bsg.ox.ac.uk/research/research-projects/covid-19-government-response-tracker and https://github.com/owid/covid-19-data/tree/master/public/data, (accessed on 12 December 2021).

## Appendix A. Definitions, Data Sources, and References of Main Variables in the Regression Equation

**Table A1:** Variable definitions, data sources, and references.

Notation	Description	Data Source	Reference
*CASES_GROW*	New case growth rate for a country *i* on a day *t*, calculated as follows: *CASES_GROW_i,t_* = (*NEW_CASES_i,t_ − NEW_CASES_i,t−_*_1_)/*NEW_CASES_i,t−_*_1_.	Our World in Data website	-
*NEW_CASES*	The count of confirmed COVID-19 cases on a certain date	Our World in Data website	-
*STRINGENCY_INDEX*	Government policy stringency index: a composite measure based on four types of governmental policies with a total of 17 indicators: (1) containment and closure policies, (2) economic policies, (3) health system policies, and (4) miscellaneous policies. The index is rescaled to a value from 0 to 100 (100 = strictest response). In particular, containment and closure policies include eight indicators, with the detailed information provided in Appendix B.	Oxford COVID-19 Government Response Tracker, Blavatnik School of Government	-
*STRINGENCY_TERCILE*	A category variable, created by splitting the sample into three subsamples based on the original stringency index of a government and assigning a measure of 1, 2, and 3 if its stringency index is in the bottom, medium, and top terciles of all sample countries on a certain day *t*, corresponding to three different strictness levels of a government’s policy response to the COVID-19 spread: below average, average, and above average.	Oxford COVID-19 Government Response Tracker, Blavatnik School of Government	-
*STRINGENCY_MEDIAN_ADJUSTED*	A country’s stringency index adjusted by all the country’s median level of the index on a day *t*.	Oxford COVID-19 Government Response Tracker, Blavatnik School of Government	-
*STRINGENCY_STANDARDIZED*	A normalized index by considering both mean and standard deviation of the original stringency index on day *t.* Note that here the day *t* refers to the *t*th day since a country reports its first case of COVID-19, not the calendar day.	Oxford COVID-19 Government Response Tracker, Blavatnik School of Government	-
*LN_POPULATION*	Natural logarithm of a country’s population in 2020	United Nations, Department of Economic and Social Affairs, Population Division, World Population Prospects: The 2019 Revision	Rubin et al. [34]
*POPULATION_DENSITY*	The number of people divided by land area, measured in square kilometers, most recent year available.	World Bank—World Development Indicators, sourced from the Food and Agriculture Organization and World Bank estimates	Hamidi et al. [33]; Rubin et al. [34]; Kadi and Khelfaoui [35]
*AGED_70_OLDER*	Share of the population that is 70 years and older in 2015.	United Nations, Department of Economic and Social Affairs, Population Division (2017), World Population Prospects: The 2017 Revision	Dowd et al. [36]; Liang et al. [15]
*LN_MEDIAN_AGE*	The median age of the population, UN projection for 2020	UN Population Division, World Population Prospects, 2017 Revision	Dowd et al. [36]; Rubin et al. [34]
*LN_GDP_PER_CAPITA*	Natural logarithm of a country’s Gross domestic product at purchasing power parity (constant 2011 international dollars), most recent year available	World Bank—World Development Indicators, source from World Bank, International Comparison Program database	Cepaluni et al. [14]
*LN_CVD_DEATH_RATE*	Natural logarithm of death rate from cardiovascular disease in 2017 (annual number of deaths per 100,000 people)	Global Burden of Disease Collaborative Network, Global Burden of Disease Study 2017 Results	Mehra et al. [41]
*DIABETES_PREVALENCE*	Diabetes prevalence (% of population aged 20 to 79) in 2017	World Bank—World Development Indicators, sourced from International Diabetes Federation, Diabetes Atlas	Rubin et al. [34]; Dowd et al. [36]
*MALE_SMOKERS*	Percentage of men who smoke, most recent year available	World Bank—World Development Indicators, sourced from the World Health Organization, Global Health Observatory Data Repository	Hamidi et al. [33]; Rubin et al. [34]
*FEMALE_SMOKERS*	Percentage of women who smoke, most recent year available	World Bank—World Development Indicators, sourced from the World Health Organization, Global Health Observatory Data Repository	Hamidi et al. [33]; Rubin et al. [34]
*HOSPITAL_BEDS_PER_100K*	Hospital beds per 100,000 people, most recent year available since 2010	OECD, Eurostat, World Bank, national government records, and other sources	Hamidi et al. [33]; Liang et al. [15]

Source: Code Book for COVID-19 data; https://github.com/owid/covid-19-data/blob/master/public/data/owid-covid-data-codebook.md#codebook-for-the-complete-our-world-in-data-covid-19-dataset, accessed on 25 September 2020.

## Appendix B. Definitions of Containment and Closure Policies Used in the Government Response Stringency Index

**Table A2:** Variable definitions of the eight containment and closure polices.

Variable	Description	Measurement	Coding
*SCHOOL_CLOSING*	Record closings of schools and universities	Ordinal scale	0—no measures 1—recommend closing 2—require closing (only some levels or categories, e.g., just high school, or just public schools) 3—require closing all levels Blank—no data
*WORKPLACE_CLOSING*	Record closings of workplaces	Ordinal scale	0—no measures 1—recommend closing (or recommend work from home) 2—require closing (or work from home) for some sectors or categories of workers 3—require closing (or work from home) for all-but-essential workplaces (e.g., grocery stores, doctors) Blank—no data
*CANCEL_PUBLIC_EVENTS*	Record canceling public events	Ordinal scale	0—no measures 1—recommend canceling 2—require canceling Blank—no data
*RESTRICTIONS_ON_* *GATHERINGS*	Record limits on private gatherings	Ordinal scale	0—no restrictions 1—restrictions on very large gatherings (the limit is above 1000 people) 2—restrictions on gatherings between 101–1000 people 3—restrictions on gatherings between 11–100 people 4—restrictions on gatherings of 10 people or less Blank—no data
*CLOSE_PUBLIC_TRANSPORT*	Record closing of public transport	Ordinal scale	0—no measures 1—recommend closing (or significantly reducing volume/route/means of transport available) 2—require closing (or prohibit most citizens from using it) Blank—no data
*STAY_AT_HOME_* *REQUIREMENTS*	Record orders to “shelter-in-place” and otherwise confined to the home	Ordinal scale	0—no measures 1—recommend not leaving the house 2—require not leaving the house with exceptions for daily exercise, grocery shopping, and ‘essential’ trips 3—require not leaving the house with minimal exceptions (e.g., allowed to leave once a week, or only one person can leave at a time, etc.) Blank—no data
*RESTRICTIONS_ON_* *INTERNAL_* *MOVEMENT*	Record restrictions on internal movement between cities/regions	Ordinal scale	0—no measures 1—recommend not to travel between regions/cities 2—internal movement restrictions in place Blank—no data
*INTERNATIONAL_TRAVEL_* *CONTROLS*	Record restrictions on international travel for foreign travelers, not citizens	Ordinal scale	0—no restrictions 1—screening arrivals 2—quarantine arrivals from some or all regions 3—ban arrivals from some regions 4—ban on all regions or total border closure Blank—no data

Source: Codebook for the Oxford Covid-19 Government Response Tracker. https://github.com/OxCGRT/covid-policy-tracker/blob/master/documentation/codebook.md#codebook-changelog accessed on 25 September 2020.
